# Presence of chest tubes does not affect the hemodynamic efficacy of standard cardiopulmonary resuscitation

**DOI:** 10.1186/s40560-017-0267-3

**Published:** 2017-12-21

**Authors:** Gu Hyun Kang, Hyun Youk, Kyoung Chul Cha, Yoonsuk Lee, Hyung Il Kim, Yong Sung Cha, Oh Hyun Kim, Hyun Kim, Kang Hyun Lee, Sung Oh Hwang

**Affiliations:** 1 Department of Emergency Medicine, Hallym University College of Medicine, Seoul, Republic of Korea; 2 Department of Emergency Medicine, Yonsei University Wonju College of Medicine, 20 Ilsanro, Wonju, Republic of Korea

**Keywords:** Cardiopulmonary resuscitation, Thoracostomy, Cardiac arrest, External chest compression, Chest tube

## Abstract

**Background:**

During cardiopulmonary resuscitation (CPR), chest tubes can hinder increases in intrathoracic pressure by venting the pressure during chest compressions, thus reducing the blood flow generated by the thoracic pump effect. The aim of the present study was to investigate the effects of chest tubes on hemodynamic efficacy during standard CPR in a swine model of cardiac arrest.

**Methods:**

Twelve domestic male pigs weighing 39.6 ± 8.4 kg underwent bilateral tube thoracostomy and received a total of 12 min of standard manual CPR, which comprised of two 6-min courses of CPR after 2 min of electrically induced ventricular fibrillation. Each 6-min set consisted of 3 min of CPR with clamped chest tubes (CCT-CPR) and 3 min of CPR with unclamped chest tubes (UCT-CPR). The sequence of CCT-CPR and UCT-CPR was randomized.

**Results:**

Hemodynamic parameters including aortic pressure, left ventricular pressure, right ventricular pressure, right atrial pressure, and minimal and maximal dp/dt did not differ significantly between CCT-CPR and UCT-CPR. No significant differences were noted in carotid blood flow, end-tidal CO_2_, or coronary perfusion pressure between CCT-CPR and UCT-CPR.

**Conclusions:**

The presence of chest tubes did not affect the hemodynamic efficacy of standard CPR. There is no need to clamp chest tubes during standard CPR.

## Background

Closed-chest cardiopulmonary resuscitation (CPR), first introduced in 1960, is now used worldwide and has been documented as effective in resuscitating cardiac arrest patients [1]. However, the mechanism of blood flow during CPR in humans has not been clearly defined. The cardiac pump theory explains that direct compression of the cardiac structure is the basic mechanism of blood flow generated by chest compressions [2–4]. On the other hand, the thoracic pump theory postulates that fluctuations of the intrathoracic pressure, not direct cardiac compressions, cause blood flow by creating a pressure gradient between the intrathoracic and extrathoracic vascular compartments [5–7]. The two mechanisms might generate blood flow with different magnitudes according to chest configuration or clinical conditions of the individual receiving CPR. The blood flow generated by standard CPR during cardiac arrest is known to be only 17–27% of the normal cardiac output [8–10]. Thus, there are concerns that any alteration of the mechanism generating blood flow during CPR could further reduce tissue perfusion.

It is not uncommon for patients to receive CPR in the presence of chest tubes due to cardiac arrest after cardiothoracic surgery or treatment of pleural diseases such as tension pneumothorax [11, 12]. The presence of chest tubes might alter the mechanism of blood flow generated by chest compressions. For instance, chest tubes might hinder increases in intrathoracic pressure by venting the pleural pressure during chest compressions, thus reducing the blood flow generated by the thoracic pump mechanism. However, little is known about the hemodynamic effects of chest compressions in the presence of chest tubes during CPR. Furthermore, current CPR guidelines do not provide any recommendations about whether chest tubes should be clamped when chest compressions are performed [13, 14].

The aim of the present study was to investigate the effects of chest tubes on hemodynamic efficacy during standard CPR in a swine model of cardiac arrest.

## Methods

### Animals and ethics

Twelve domestic male pigs weighing 39.6 ± 8.4 kg from a single-source breeder were used in this study. The experimental procedures and protocols conformed to the institutional guidelines for the care and use of animals in research and were approved by the Institutional Animal Care and Use Committee of Wonju College of Medicine, Yonsei University (YWC-140408).

### Animal preparation

The animals were fasted overnight but allowed free access to water. Anesthesia was initiated by the intramuscular injection of ketamine (20 mg/kg) and maintained by ear vein injection of ketamine (30 mg/kg). Body temperature was maintained between 36.5 and 37.5 °C during the procedures, using an incandescent heat lamp and electric heat pad. After anesthesia, endotracheal intubation was performed with a cuffed endotracheal tube. Intubation was confirmed based on the endotracheal end-tidal carbon dioxide concentration (EtCO_2_) (CO2SMO, Phillips Respironics, Murrysville, PA, USA). After intubation, the pigs were placed in the supine position. The animals were ventilated with room air via a volume-controlled ventilator (MDS Matrix 3000, Orchard Park, NY, USA) during preparation. The tidal volume was set at 10 mL/kg, and the ventilation rate was set at 18 breaths per minute. Electrocardiography (ECG lead II) and EtCO_2_ were monitored continuously.

Under aseptic conditions, the right femoral artery was cannulated with an introducer sheath (7.5 Fr, Arrow International Inc., Reading, PA, USA) by the Seldinger method, and the aortic blood pressure was recorded continuously with a micromanometer-tipped catheter (5 Fr., Millar Instruments, Inc., Houston, TX, USA) introduced into the femoral artery. After the right cervical dissection, the right carotid artery was cannulated with an introducer sheath (7.5 Fr) by the Seldinger method, and the left ventricle (LV) pressure was recorded continuously with a micromanometer-tipped catheter (5 Fr., Millar Instruments, Inc., Houston, TX, USA) introduced into the right carotid artery. Two introducer sheaths were placed in the right external jugular vein—one recorded the right atrium (RA), and the other the right ventricle (RV) pressure via a micromanometer-tipped catheter (6 Fr., Millar Instruments, Inc., TX, USA). After the left cervical dissection, the left carotid artery was surgically exposed, and an ultrasonic flow probe (T106, Transonic Systems Inc., Ithaca, NY) was placed around it to quantify blood flow. The catheter position was confirmed by characteristic pressure tracing from the cardiac chamber and by postmortem examination. Once the catheters were in place, a heparin bolus (100 u/kg, IV) was administered to prevent thrombosis.

Chest tubes (16 Fr) were inserted in both the mid-lateral thoracic walls. A micromanometer-tipped catheter (5 Fr) was inserted into the thoracic space via the left chest tube to record the intrapleural pressure (IPP). The chest tubes were clamped with a curved hemostat without teeth after insertion.

### Induction of ventricular fibrillation and CPR

After baseline data were collected, a pacing catheter (5 Fr, bipolar lead, Arrow International Inc., Reading, PA, USA) was positioned in the RV. For the induction of ventricular fibrillation (VF), an electrical current at 60 Hz was delivered to the endocardium. VF was confirmed by the ECG waveform and a decline in aortic pressure. Once VF was induced, the endotracheal tube was disconnected from the ventilator, and the pigs were observed for 2 min without any procedure or treatment.

After 2 min of VF, the animals received a total of 12 min of standard manual CPR. Chest compressions were performed by experienced emergency medical technicians who had passed the American Heart Association (AHA) Basic Life Support (BLS) Provider Course. Chest compressors were blinded to the study design. Two chest compressors performed chest compressions every 2 min according to BLS guidelines [15]. The compression and ventilation ratio was 30:2, and the compressors were switched every 2 min. Chest compressions were performed at a depth of 5 cm and a rate of 100/min. Positive pressure ventilations were delivered with a resuscitator bag (silicone resuscitator 870040, Laerdal Medical, Stavanger, Norway) every 30 chest compressions.

### Experimental protocol

The animals received a total of 12 min of standard manual cardiopulmonary resuscitation (CPR), which comprised two 6-min courses of CPR. Each 6-min CPR set consisted of 3 min of CPR with clamped chest tubes (CCT-CPR) and 3 min of CPR with unclamped chest tubes (UCT-CPR). The experiment was designed as a crossover trial. The sequence of CCT-CPR and UCT-CPR was randomized according to a schedule enclosed in a sealed envelope. For animals allocated to protocol A, both chest tubes were clamped (CCT-CPR) for the first 3 min and unclamped (UCT-CPR) for the next 3 min during CPR. For animals allocated to protocol B, both chest tubes were unclamped (UCT-CPR) during the first 3 min and clamped (CCT-CPR) for the next 3 min during CPR. Each sequence was performed twice, for a total of 12 min of CPR.

### Data measurements

Data were digitized with a digital recording system (Powerlab, AD Instruments, Colorado Springs, CO, USA). All parameters (aortic, RV, RA, and LV systolic and diastolic pressures; left pleural pressure; carotid blood flow; and EtCO_2_) were continuously recorded and analyzed at the baseline. The coronary perfusion pressure (CPP) during CPR was calculated as the difference between the aortic pressure and the RA pressure in the end-diastolic phase using an electronic subtraction unit. The rates of increase in LV and RV pressure were measured as the dp/dt (minimum and maximum). The dp/dt of the LV and RV was calculated from the pressure tracings with a program from a digital recording system.

### Statistical analysis

For hemodynamic data analysis, the average value of each parameter measured during the data sampling period was used. Data are expressed according to the properties of the variables. Continuous variables are presented as the mean and standard deviation after normality assessment by the Shapiro-Wilk test. A two-sample *t* test or analysis of variance (ANOVA) was used to compare continuous variables as appropriate. Analyses were performed with SPSS V.23.0 software (IBM Corp., Chicago, IL, USA). Differences were regarded as significant if the *p* values were less than 0.05.

## Results

### Baseline measurements of animals

Among the 12 animals initially included in the study, two suffered VF during catheterization. Therefore, ten animals were included in the final analysis. The mean body weight was 39 ± 8 kg, and the mean chest circumference was 69 ± 6 cm. No differences in baseline measurement (body weight, chest circumference, rectal temperature, and baseline oxygen saturation) were noted between the animals allocated to protocol A or B. There were no significant differences in baseline hemodynamic measurements between the animals allocated to protocol A or B (Table 1).

**Table 1:** Comparison of the baseline hemodynamic measurements between animals allocated to protocol A or B

Parameter	Animals of protocol A (*n* = 5)	Animals of protocol B (*n* = 5)	*p* value
Systolic aortic pressure (mmHg)	124 ± 4	120. ± 9	0.36
Diastolic aortic pressure (mmHg)	92 ± 8	82 ± 8	0.10
Systolic LV pressure (mmHg)	113 ± 9	116 ± 21	0.76
Diastolic LV pressure (mmHg)	7 ± 7	5 ± 4	0.46
Systolic RV pressure (mmHg)	21 ± 7	24 ± 20	0.80
Diastolic RV pressure (mmHg)	2 ± 1	2 ± 2	0.75
Mean RA pressure (mmHg)	3 ± 2	3 ± 3	0.93
dp/dt RV max. (mmHg/s)	143 ± 48	190 ± 102	0.38
dp/dt RV min. (mmHg/s)	− 135 ± 32	− 155 ± 37	0.40
dp/dt LV max. (mmHg/s)	435 ± 198	551 ± 343	0.54
dp/dt LV min. (mmHg/s)	− 445 ± 203	− 461 ± 154	0.89
CBF (mL/min)	232 ± 147	198 ± 83	0.67
EtCO_2_ (mmHg)	45 ± 6	44 ± 5	0.66

All variables are shown as mean ± standard deviation. Baseline indicates measurements before induction of cardiac arrest

*RA* right atrium, *RV* right ventricle, *LV* left ventricle, *max.* maximum, *min.* minimum, *CBF* carotid blood flow, *EtCO*_*2*_ end-tidal CO_2_ concentration

### Effects of chest tubes on hemodynamic parameters during CPR

Chest tubes did not affect hemodynamic parameters during CPR. Systolic hemodynamic parameters including aortic systolic pressure, left ventricular systolic pressure, right ventricular systolic pressure, and right atrial systolic pressure did not differ significantly between CCT-CPR and UCT-CPR. No significant differences were noted in diastolic hemodynamic parameters including aortic diastolic pressure, left ventricular diastolic pressure, right ventricular diastolic pressure, and right atrial diastolic pressure between CCT-CPR and UCT-CPR. The rates of increase in LV and RV pressure (measured by the maximal dp/dt) did not differ between CCT-CPR and UCT-CPR. The rates of decrease in LV and RV pressure (measured by the minimal dp/dt) also did not differ between CCT-CPR and UCT-CPR (Table 2).

**Table 2:** Comparison of systolic and diastolic parameters between CCT-CPR and UCT-CPR

Parameter (mmHg)	CCT-CPR for 1st 3 min (*n* = 10)	UCT-CPR for 1st 3 min (*n* = 10)	*p* value	CCT-CPR for 2nd 3 min (*n* = 10)	UCT-CPR for 2nd 3 min (*n* = 10)	*p* value
Systolic aortic pressure (mmHg)	117 ± 69	105 ± 53	0.572	124 ± 161	104 ± 52	0.895
Diastolic aortic pressure (mmHg)	9 ± 8	11 ± 7	0.106	9 ± 8	11 ± 8	0.020
Systolic LV pressure (mmHg)	183 ± 72	164 ± 69	0.558	168 ± 77	162 ± 71	0.324
Diastolic LV pressure (mmHg)	9 ± 6	10.5 ± 7	0.102	8 ± 7	9 ± 7	0.229
Systolic RV pressure (mmHg)	184 ± 89	157 ± 83	0.075	171 ± 90	158 ± 85	0.032
Diastolic RV pressure (mmHg)	12 ± 7	12 ± 6	0.813	12 ± 6	12 ± 8	0.808
Systolic RA pressure (mmHg)	174 ± 87	149 ± 79	0.086	162 ± 91	151 ± 83	0.097
Diastolic RA pressure (mmHg)	11 ± 6	12 ± 6	0.658	11 ± 6	12 ± 6	0.333
dp/dt RV max. (mmHg/s)	747 ± 580	641 ± 500	0.544	698 ± 595	659 ± 545	0.162
dp/dt RV min. (mmHg/s)	− 657 ± 397	− 571 ± 354	0.781	− 603 ± 382	− 582 ± 370	0.313
dp/dt LV max. (mmHg/s)	751 ± 532	641 ± 500	0.813	687 ± 558	674 ± 508	0.808
dp/dt LV min. (mmHg/s)	− 681 ± 321	− 571 ± 354	0.453	−611 ± 325	− 615 ± 316	0.965

All variables are shown as mean ± standard deviation. Baseline indicates measurements before induction of cardiac arrest

*CCT-CPR* cardiopulmonary resuscitation with clamped chest tubes, *UCT-CPR* cardiopulmonary resuscitation with unclamped chest tubes, *RA* right atrium, *RV* right ventricle, *LV* left ventricle, *max.* maximum, *min.* minimum

### Intrapleural pressure

The mean IPP was significantly lower during spontaneous circulation than during CCT-CPR and UCT-CPR (*p* = 0.003). However, chest tubes did not affect the IPP. No significant differences in the IPP variables were noted between CCT-CPR and UCT-CPR (Table 3).

**Table 3:** Comparison of intrapleural pressure between CCT-CPR and UCT-CPR

Parameter	CCT-CPR for 1st 3 min (*n* = 10)	UCT-CPR for 1st 3 min (*n* = 10)	*p* value	CCT-CPR for 2nd 3 min (*n* = 10)	UCT-CPR for 2nd 3 min (*n* = 10)	*p* value
IPP max. (mmHg)	17 ± 22	12 ± 11	0.586	16 ± 23	10 ± 13	0.531
IPP min. (mmHg)	− 9 ± 6	− 6 ± 5	0.141	− 8 ± 6	− 13 ± 7	0.857
IPP mean (mmHg)	0 ± 4	1 ± 6	0.975	1 ± 5	1 ± 6	0.517
IPP gradient (mmHg)	26 ± 25	19 ± 9	0.441	24 ± 26	11 ± 8	0.15

All variables are shown as mean ± standard deviation

*IPP* intrapleural pressure, *CCT-CPR* cardiopulmonary resuscitation with clamped chest tubes, *UCT-CPR* cardiopulmonary resuscitation with unclamped chest tubes, *max.* maximum, *min.* minimum

### Carotid blood flow, end-tidal carbon dioxide concentration, and coronary perfusion pressure

No significant differences were noted in the carotid blood flow, EtCO_2_, or CPP between CCT-CPR and UCT-CPR (Table 4).

**Table 4:** Comparison of coronary blood flow, end-tidal carbon dioxide concentration, and coronary perfusion pressure between CCT-CPR and UCT-CPR

Parameter	CCT-CPR for 1st 3 min (*n* = 10)	UCT-CPR for 1st 3 min (*n* = 10)	*p* value	CCT-CPR for 2nd 3 min (*n* = 10)	UCT-CPR for 2nd 3 min (*n* = 10)	*p* value
EtCO_2_ (mmHg)	40 ± 10	34 ± 6	0.662	31 ± 5	38 ± 11	0.215
CBF (mL/min)	143 ± 151	158 ± 94	0.851	159 ± 107	103 ± 113	0.439
CPP (mmHg)	12 ± 5.2	16 ± 5	0.252	13 ± 7	9 ± 5	0.410

All variables are shown as mean ± standard deviation

*EtCO*_*2*_ end-tidal carbon dioxide concentration, *CBF* carotid blood flow, *CPP* coronary perfusion pressure, *CCT-CPR* cardiopulmonary resuscitation with clamped chest tubes, *UCT-CPR* cardiopulmonary resuscitation with unclamped chest tubes

## Discussion

Our study demonstrates that there are no differences in hemodynamic parameters including pressures and pressure changes rates of the ventricles, carotid blood flow, or coronary perfusion pressure between CPR with chest tubes and standard CPR (CPR with clamped chest tubes). This finding suggests that it is unnecessary to clamp the chest tubes when CPR is performed in cardiac arrest patients with chest tubes. This is the first study to investigate the hemodynamic effects of chest tubes during CPR.

Chest tubes are inserted into the pleural cavity to drain air, blood, or fluid in patients who have pneumothorax or hemothorax or have undergone cardiothoracic surgery. Chest tubes should be connected to an underwater sealed drainage system to prevent backflow of air or fluid to the pleural cavity and thus maintain adequate respiratory function [16]. There is a high risk for cardiac arrest in a substantial proportion of patients with chest tubes. Perioperative cardiac arrest, which is associated with the high cardiac surgical mortality, occurs in 5.2–5.5% of patients undergoing cardiothoracic operations [17, 18]. When a patient with chest tubes is resuscitated, the presence of the chest tubes might alter the hemodynamic efficacy by exposing the intrapleural space to the atmosphere. The phasic rise of intrathoracic pressure or simultaneous lung inflation during chest compressions is known to augment cerebral and coronary perfusion [7, 19]. A chest tube might act as a vent that prevents this increase in intrathoracic pressure and thus might reduce the thoracic pump effect that generates blood flow during chest compressions. If this is true, should chest tubes be clamped to maintain adequate hemodynamic effects during CPR? Our study provides the answer to this question.

In our study, unclamping the chest tubes during CPR did not alter hemodynamic parameters such as the systolic and diastolic pressures of the cardiac chambers, the coronary perfusion pressure, and the rates of pressure change measured by the dp/dt of the ventricles. Surrogates of blood flow to the brain and pulmonary circulation measured by carotid blood flow and end-tidal CO_2_ tension were not altered when the chest tubes were unclamped. Unclamping the chest tubes during CPR also did not significantly alter the intrapleural pressure. This finding indicates that the chest tubes might be collapsed or sealed by the visceral pleura during the compression phase of CPR, thus preventing the venting of intrathoracic pressure. This needs to be investigated further. Unclamped chest tubes might produce a pneumothorax and thus reduce the venous return and blood flow generated by chest compressions. Exposure of the intrapleural space to the atmosphere through unclamped chest tubes would cause pneumothorax if spontaneous breathing were occurring. However, positive-pressure ventilation is performed during CPR so that persistent negative intrathoracic pressure does not develop. The maximal and minimal intrapleural pressure did not differ between UCT-CPR and CCT-CPR in this study. This demonstrates that the venting effect of unclamped chest tubes is negligible or minimal during CPR. Furthermore, the chest tubes are connected to an underwater sealed drainage system in clinical settings, so there is little possibility of hemodynamic alteration due to the chest tubes during CPR.

Chest compressions can cause parenchymal lung injuries including pneumothorax [20, 21]. Clamping of the chest tubes during CPR might increase the risk of tension pneumothorax when an unnoticed pneumothorax develops due to chest compressions. In this context, it is reasonable to keep the chest tubes unclamped during CPR.

### Limitations

This study has some limitations. Our study protocol involved a crossover trial from UCT-CPR to CCT-CPR or vice versa every 3 min in each animal. Due to the nature of the study design, an order effect might have occurred. We randomized the order of CPR to minimize the order effect. Also, we placed a micromanometer-tipped catheter in the pleural cavity via the chest tube of the left thorax to measure the intrapleural pressure. These data might not have exactly matched the actual intrapleural pressures of both pleural spaces because we only measured the pleural pressure from the unilateral thorax. In this experiment, low CPP was observed because our experimental protocol did not include epinephrine administration to simulate BLS situation. However, maintaining adequate CPP over 20 mmHg is important to restore spontaneous circulation and cerebral oxygenation, so that low CPP observed in this experiment can be a limiting factor to apply our result to clinical setting [22]. Finally, this was an animal experimental study to simulate CPR in the presence of chest tubes. In the clinical setting, the patient with chest tubes might have additional clinical conditions which need thoracostomies such as pneumothorax, hemothorax, or surgical procedures of the thorax. Therefore, caution is needed when interpreting and applying these results in clinical settings.

## Conclusion

The presence of chest tubes did not affect the hemodynamic efficacy of standard CPR in an animal model of cardiac arrest. There is no need to clamp the chest tubes during standard CPR.
